# Relating experimentally-induced fear to pre-existing phobic fear in the human brain

**DOI:** 10.1093/scan/nsx147

**Published:** 2017-12-21

**Authors:** Seth M Levine, Michael Pfaller, Jonas Reichenberger, Youssef Shiban, Andreas Mühlberger, Rainer Rupprecht, Jens V Schwarzbach

**Affiliations:** 1 Department of Psychiatry and Psychotherapy, University of Regensburg, 93053 Regensburg, Germany; 2 Department of Psychology (Clinical Psychology and Psychotherapy), University of Regensburg, 93053 Regensburg, Germany

**Keywords:** cross-decoding, fear-conditioning, fMRI, phobia, similarity analysis

## Abstract

While prior work has demonstrated that fear-conditioning changes the neural representation of previously neutral stimuli, it remains unknown to what extent this new representation abstracts away from specific fears and which brain areas are involved therein. To investigate this question, we sought commonalities between experimentally-induced fear via electric shocks and pre-existing phobia. Using functional MRI, we tested the effect of fear-conditioning pictures of dogs in 21 spider-fearful participants across three phases: baseline, post-conditioning, and extinction. Considering phobic stimuli as a reference point for the state of fear allowed us to examine whether fear-conditioning renders information patterns of previously neutral stimuli more similar to those of phobic stimuli. We trained a classification algorithm to discriminate information patterns of neutral stimuli (rats) and phobic stimuli and then tested the algorithm on information patterns from the conditioned stimuli (dogs). Performing this cross-decoding analysis at each experimental phase revealed brain regions in which dogs were classified as rats during baseline, as spiders following conditioning, and again as rats after extinction. A follow-up analysis showed that changes in visual perception information cannot explain the changing classification performance. These results demonstrate a common neural representation for processing fear-eliciting information, either pre-existing or acquired by classical conditioning.

## Introduction

Survival depends on an organism’s ability to avoid threats through fear learning. The classic concept of Pavlovian fear conditioning assumes that a previously neutral stimulus can elicit a fear response from an organism if that stimulus has been associated with an aversive, unconditioned stimulus (US) (Fendt and Fanselow, 1999; Watson and Rayner, 1920). A large body of studies that used functional magnetic resonance imaging (fMRI) has discovered brain regions in which a conditioned stimulus (CS+) evokes a systematically stronger or weaker blood-oxygen-level dependent (BOLD) signal than a neutral stimulus (NS) (Andreatta *et al.*, 2012; Bach *et al.*, 2011; Büchel *et al.*, 1998; Fullana *et al.*, 2016; Gross and Canteras, 2012; Holland and Bouton, 1999; LaBar *et al.*, 1998). More recently, pattern-based analyses of fear-conditioning have shown that, after conditioning, representations of stimuli that are fear-conditioned and belong to the same visual category become more similar to each other (Dunsmoor *et al.*, 2014), and that fear-conditioned stimuli become more similar to the US (Onat and Büchel, 2015). However, in such cases, the reference point for fear has been a concrete aversive stimulus (i.e. the US), such as an electric shock. Thus, discovering increased similarity within a fear-conditioned category or between a CS+ and the US raises the question as to whether this similarity pertains to abstract properties of fear or to the association with a concrete aversive stimulus (for example, an electric shock as the US would be present in both the CS+ condition and the US condition).

**Fig. 1.**
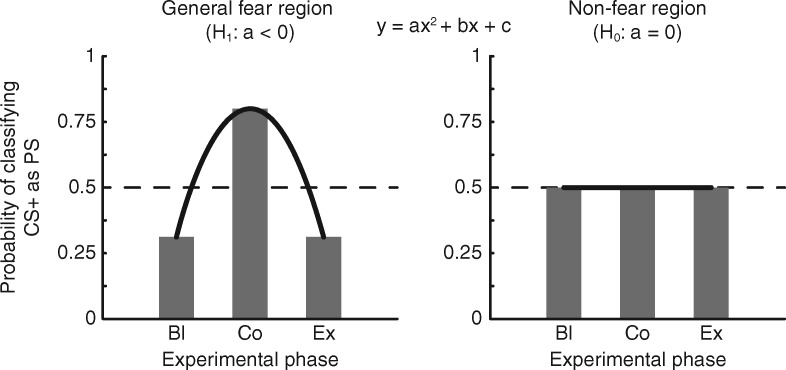
Predicted results: regions whose underlying mechanisms pertain to general fear processing should influence a machine learning algorithm to classify a conditioned stimulus (CS+) as similar to a phobic stimulus (PS) after fear-conditioning (Co). However, during baseline (Bl) and following fear-extinction (Ex), the classifier should consider the CS+ as similar to another neutral stimulus (NS). Using a quadratic regression (dark gray curves), we expect regions exhibiting these properties to yield an inverted-U shape (quadratic coefficients that are less than zero), while regions that play no role should yield a flat profile (quadratic coefficients that approach zero).

**Fig. 2.**
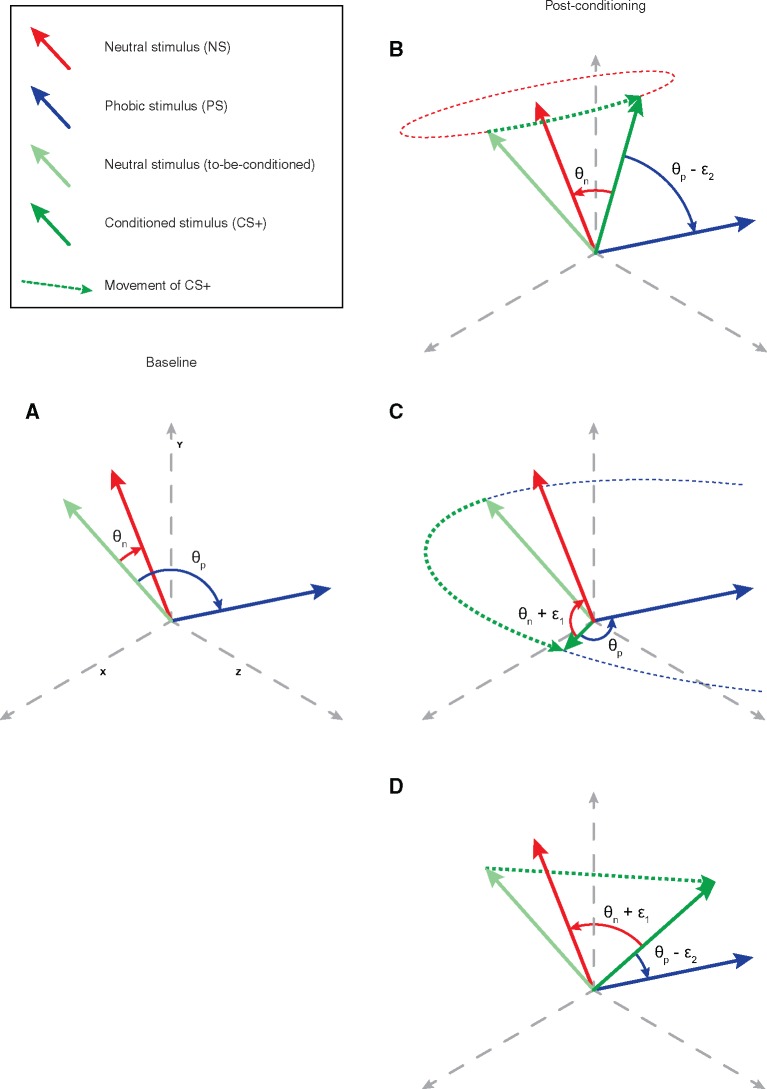
Depiction of three models that can underlie the results of the decoding analysis. One can measure similarity as the angle between vectors in a multi-dimensional space. Measuring the similarity in the baseline phase (A) between the neutral stimulus (NS; red vector and the to-be-conditioned NS (light-green vector) yields angle θ_n_, while measuring the similarity between the to-be-conditioned NS and the phobic stimulus (PS; blue vector) yields angle θ_p_. After conditioning, the first model (B) describes the scenario in which the conditioned stimulus (CS+; dark-green vector) becomes more similar to the PS but does not change with respect to the NS, which is depicted by the CS+ moving (dotted green line) toward the PS but in a circular trajectory around the NS (dotted red line), resulting in θ_p_ decreasing by some non-zero quantity (ɛ_2_) while θ_n_ remains the same. The second model (C) describes the converse scenario: the CS+ becomes less similar to the NS but does not change with respect to the PS. This is depicted by the CS+ moving further away from the NS but in a circular trajectory around the PS (dotted blue line), resulting in θ_p_ remaining the same while θ_n_ increases by some non-zero quantity (ɛ_1_). The third model (D) describes the combined scenario: the CS+ becomes less similar to the NS and more similar to the PS, in which case the CS+ moves away from the NS and toward the PS, resulting in θ_n_ increasing by ɛ_1_ and θ_p_ decreasing by ɛ_2_.

Therefore, in order to study the state of fear independent of the effect of a concrete US, we compared neural representations of CS+’s (which were experimentally paired with electric shocks) to neural representations of pre-existing phobic fear of spiders (which were not paired with electric shocks). To this aim, we recruited 21 spider-fearful participants and used fMRI to measure their BOLD response while they viewed pictures of spiders, dogs, and rats. During the experiment, participants acquired a fear of dogs (CS+) by means of mild, unpleasant electric shocks (US) to their wrist. Pictures of rats served as neutral control stimuli (NS). By using neural patterns elicited by pictures of spiders as the reference point for the state of fear, we combined a whole-brain searchlight analysis (Kriegeskorte *et al.*, 2006) with a linear discriminant analysis classifier to discover how a classifier that learned to discriminate the NS (rats) from the PS (spiders) would classify the CS+ (dogs) in different experimental phases (i.e. prior to fear-conditioning, after fear-conditioning, and following fear-extinction). Brain areas that represent whether a participant fears a stimulus should initially classify dogs (still NS) as rats (NS), i.e., as emotionally neutral. After fear conditioning, such areas should classify the dogs (now CS+) as spiders (PS), because now both animal classes induce fear. In the extinction phase, when participants do not fear dogs anymore, dogs should be classified in these areas as rats (NS) again. In summary, areas that represent the emotional content of a stimulus should show an inverted quadratic timecourse of the probability of classifying dogs as spiders (low, high, low) over the experimental phases baseline, conditioned, and extinction (see Figure 1). We followed up this primary analysis with two subsequent analyses whose aims were to determine (i) whether similarity changes between CS+ and PS or between CS+ and NS drove the classifier’s differential performance in the revealed regions (see Figure 2 and *Material and Methods: Similarity Analysis*) and (ii) whether we could rule out changes in similarity of visual perception information as a means of explaining the primary results (see *Materials and Methods: Region of Interest Analysis*).

## Materials and methods

### Participants and questionnaires

Potential participants were screened using a German online questionnaire, which reflects the four central diagnostic criteria for specific phobia in DSM-5 on a scale from 0 to 6 (Rinck *et al.*, 2002), and equivalently worded questionnaires for fear of dogs and rats. Only participants with responses of greater than or equal to 5 on the dimensions ‘fear’ and ‘arousal’ for spiders and less than or equal to 3 on these dimensions for dogs and rats were contacted via email. The second round of screening consisted of responding to the German version (Rinck *et al.*, 2002) of the ‘Fear of Spiders Questionnaire’ (Szymanski and O’Donohue, 1995), in order to better quantify participants’ fear of spiders, and two derivative questionnaires to additionally quantify their fear of dogs and rats on 0 to 6 point scales. We invited participants to take part in the study if they scored greater than or equal to 44 (i.e. average score of ∼3 per item) in the questionnaire and less than an average score of 3 per item on the rat and dog questionnaires. Additional exclusion criteria were past/present treatment for mental or neurological disorders, present intake of psychotropic medication, pregnancy, and contraindications to MR scanning. Included participants (21 females, age range = 19–30 years) yielded mean scores for spiders (median = 3.78; IQR = 3.40–4.40) that were higher than that of dogs, assessed with Wilcoxon signed-rank tests, (median = 0.13; IQR = 0–0.28; W = 231, z = 4.0145, *P* < 5.957 × 10^−5^) and of rats (median = 0.75; IQR = 0.38–1.16; W = 231, z = 4.0148, *P <* 5.9493 × 10^−5^). A further participant took part in the experiment but was excluded from all analyses after reporting having not experienced the electroshocks as unpleasant. Experimental procedures followed safety guidelines for MRI research at the University of Regensburg, complied with the Declaration of Helsinki, and were approved by the local ethics committee.

### Stimuli

Experimental stimuli were images of dogs, spiders, and rats on a uniform gray background. A set of 26 participants (19 females, 7 males; age range = 20–39 years) who did not take part in the neuroimaging experiment rated the images for their valence and arousal using a procedure derived from Bradley and Lang (Bradley and Lang, 1994). Spider stimuli included in the main experiment were those images with the greatest negative valence and arousal values, while included dog and rat images were those with the smallest cross-category Euclidean distance in a 2 D valence-arousal coordinate plane.

### Stimulus presentation

Visual stimulation was carried out using A Simple Framework (ASF) (Schwarzbach, 2011), built on the Psychophysics toolbox (Brainard, 1997; Pelli, 1997), and MATLAB R2015b (The Mathworks, Natick, USA). An LCD video projector (JVC DLA-G20, Yokohama, Japan) cast visual stimuli behind participants in the MR scanner onto a semitransparent screen at a frame rate of 60 Hz and a resolution of 1024 × 768 pixels. Participants viewed stimuli, which subtended a visual angle of ∼13°, via a mirror positioned on the head coil. Electric shocks (duration = 2 ms) were delivered using an MR compatible DS7A current stimulator (Digitimer Limited, Letchworth Garden City, UK), the timing of which was controlled through ASF.

### Experimental design

Prior to the main experimental session, participants engaged in a threshold acquisition session, in which they shocked their right wrists starting with low current stimulation (i.e. 1 mA), increasing the amperage until they found an intensity that produced a near-painful sensation, which they deemed very unpleasant but bearable. This individualized amperage (range = 2–9 mA) was used in the main experiment during the conditioning and conditioned runs.

The experiment followed a two-factorial design with factors experimental phase [baseline, conditioned, extinction] × stimulus class [conditioned (CS+), neutral (NS), phobia (PS)]. Experimental sessions followed an event-related design and were organized into 8 runs: 2 baseline runs, 1 conditioning run, 3 conditioned runs, and 2 extinction runs. Neuroimaging data acquired during the conditioning run were not used in any further analyses. Each run (except the conditioning run) contained 36 trials (i.e. two repetitions of six images per class) pseudorandomized such that no condition appeared more than twice in a row. A given trial contained a central, green fixation dot of 2 s, followed by the presentation of an animal image for 1.5 s, followed by a temporally jittered intertrial interval of 6 + X s, with X∼geom(0.3), truncated at 10 s, during which the fixation dot was red. During 4 of the 12 CS+ trials (i.e. ∼33%) in each conditioned run, a single electric shock was administered at the offset of the stimulus. Before the extinction phase, we removed the electrostimulator from the participants’ wrist to accelerate extinction.

The conditioning run, which did not include the presentation of stimuli from the phobia class (i.e. images of spiders), contained 24 trials (i.e. two repetitions of six images per presented class) that followed the same timing scheme as those of the other runs, with the exception that the interval was temporally jittered intertrial interval of 6 + X s, with X∼geom(0.3 s), truncated at 8 s to save time. Moreover, during 6 of the 12 CS+ trials (i.e. 50%) in the conditioning run, electric shocks were administered at the offset of the stimulus.

### Neuroimaging data acquisition

Data acquisition was carried out using a 3 T Allegra head scanner (Siemens, Erlangen, Germany). Functional images were acquired with a T2*-weighted EPI sequence [34 slices per volume in ascending interleaved order, Field of view (FOV) = 64 × 64 mm^2^, voxel resolution (VR) = 3 mm^3^ isotropic, repetition time (TR) = 2000 ms, echo time (TE) = 30 ms, flip angle (FA) = 90°, gap-size = 16%, pixel bandwidth (BW) = 2790 Mhz].

For coregistration of the functional images to high-resolution anatomical images, we acquired 160 axial slices of a T1-weighted scan using a Turboflash MPRAGE sequence (FOV = 240 × 256 mm^2^, VR = 1 mm^3^ isotropic, TR = 2500 ms, TE = 2.6 ms, FA = 9°, pixel BW = 900 Mhz) for each participant.

### Neuroimaging data analysis

Analysis of the acquired neuroimaging data was carried out with the FMRIB Software Library (FSL) (Smith *et al.*, 2004) and the CoSMoMVPA toolbox (Oosterhof *et al.*, 2016) for MATLAB.

### Pre-processing

At the beginning of each functional scan, we acquired three dummy volumes to account for signal saturation. Pre-processing of the functional data included slice time correction, motion correction with respect to the middle volume of each run (using 6 degrees of freedom and trilinear interpolation), and high-pass filtering (cutoff = 100 s). For each participant, functional data were then co-registered to the corresponding high-resolution structural scan in native space using 7 degrees of freedom (Jenkinson *et al.*, 2002). In order to visualize group-level statistics, an additional co-registration to a standard MNI structural scan was performed using 12 degrees of freedom. For technical reasons, we were unable to obtain a structural scan from one participant; her functional data were co-registered directly to the standard MNI structural scan.

### Multivariate pattern analysis

To answer our first question of whether conditioned stimuli inherit properties of phobic stimuli, we performed multivariate pattern analysis (MVPA) (Haxby *et al.*, 2001), a technique for revealing information spread across multiple voxels rather than looking at each voxel individually. We carried out a whole-brain searchlight (Kriegeskorte *et al.*, 2006), which, voxel-by-voxel, restricts the analysis to local patterns surrounding the current searchlight's central voxel.

The inputs to the classifier were *t*-score maps resulting from single-trial general linear models (GLM; from the beta-weights resulting from the GLM). Hemodynamic response functions (HRFs) were modeled by convolving regressors of interest, which were all combinations of the experimental phase and stimulus class with gamma functions using FSL’s default parameters (ϕ = 0 s, σ = 3 s, mean lag = 6 s). Motion correction parameters for six dimensions (3 translations, 3 rotations) were modeled as regressors of non-interest. No spatial smoothing was applied to the functional data. Trials in which participants received electric shocks (i.e. ∼33% of trials during conditioned runs) were modeled separately from the CS+ trials in which no electric shocks were administered.

Within our whole-brain maps, we ran a 50-voxel volumetric searchlight analysis (Kriegeskorte *et al.*, 2006), in which a linear discriminant analysis classifier (LDAC) learned to distinguish patterns of *t*-scores of the NS condition from those of the PS condition (pooled from all phases of the experiment); we then tested the LDAC on patterns of *t*-scores from the CS+ condition (independently for each phase of the experiment). The train/test scheme followed a leave-one-run-out cross-validation procedure, such that, e.g., if test samples were from run 1, then training samples were from runs 2 through 7, etc. This way, no run-based effects influenced the classifier’s performance. The resulting value of a given voxel was the average of that voxel from all folds of the given experimental phase and indicated the probability of the LDAC classifying a test sample of the CS+ as the phobia class.

In order to statistically demonstrate changes in the classifier’s performance across the three experimental phases, we carried out a quadratic regression at each voxel for each participant yielding whole-brain maps of quadratic coefficients (see Figure 1). We tested coefficients for non-normality by randomly sampling (without replacement) 10 000 voxels from the group-concatenated dataset and applying an Anderson-Darling test to each vector of coefficients, which failed to find departures from normality in 94.9% of voxels. One-sample *t*-tests against zero (one-tailed, as our alternative hypothesis predicted negative quadratic coefficients) were performed on these coefficients, and the resulting *t*-score maps were corrected for multiple comparisons using 10 000 iterations of a Monte Carlo resampling procedure, in which, on a given iteration, we drew, without replacement, a random number of participants, flipped the sign of those participants coefficient maps (Nichols and Holmes, 2002), recomputed the *t*-score across participants, calculated the threshold-free cluster enhancement (TFCE) (Smith and Nichols, 2009) scores (using default parameters: E = 0.5, H = 2, dh = 0.1) from the *t*-map, and stored the map’s largest TFCE scores (to correct for the family-wise error rate), to create a null distribution for hypothesis testing of the TFCE score observed from our original *t*-score map. Empirical *P*-values were derived from the null distribution by computing the sum of the null TFCE scores that were greater than or equal to our observed TFCE score divided by the number of resampling iterations (and adding 1 to both the numerator and denominator).

The resulting multiple-comparisons-corrected statistical map was thresholded at z = 2.5758 (*P <* 0.005, family-wise error rate corrected, for improved spatial specificity of cluster definition), from which contiguous voxels containing surviving *t*-scores yielded clusters whose anatomical labels were determined by the location of each cluster’s peak value within the Harvard–Oxford cortical and subcortical structural atlases (Desikan *et al.*, 2006; Frazier *et al.*, 2005; Goldstein *et al.*, 2007; Makris *et al.*, 2006).

### Similarity analysis

In areas in which the classifier’s performance changed as a function of fear-conditioning and fear-extinction, the similarity of activity patterns between NS, CS+, and PS classes must have changed. In order to understand which changes in the representational space were potentially underlying the LDAC’s performance (see Figure 2), we calculated the similarity based on the angle between different conditions’ vector representations (Kriegeskorte *et al.*, 2008). The reason for using the similarity analysis as a follow-up, rather than as the primary analysis, was to allow the machine learning algorithm to determine when an effect size was sufficiently large to classify CS+ as PS rather than as NS. Using only inferential statistics based on similarity analyses could lead to extremely small effect sizes (that nevertheless survive statistical thresholds; e.g. consistent changes of 1° in the angle between baseline vectors and post-conditioning vectors), which would be rather difficult to interpret within the context of our hypotheses. From the spherical neighborhoods around the peak voxels of the previously discovered regions, we obtained the average patterns of the NS and PS conditions (again, pooled across experimental phases) and the average pattern of the CS+ condition at each experimental phase. Then we detrended the patterns and measured the cosine between each CS+ average and the other conditions’ averages, which is defined as

$$\cos\;\theta =\frac{\vec{u}\cdot \vec{v}}{\left\|\left.\left\|\vec{u}\right\|\right\|\right.\cdot \left\|\left.\left\|\vec{v}\right\|\right\|\right.}$$

where u and v are vectors (denoted by the arrows), ∥u∥ indicates the magnitude of vector u, and · represents the dot product of two vectors. The cosine’s output is the interval [–1, 1], where 1 indicates similarity, 0 indicates independence, and –1 indicates opposition.

### Correlation analysis

In order to quantify which of the changing similarities were possibly driving the LDAC’s differential performance, we Fisher transformed the resulting values from the similarity analysis and the decoding analysis [after rescaling to (−1, 1)] before using Pearson’s *r* to correlate the cos(∠_NS, CS+_) results and the cos(∠_PS, CS+_) results with the LDAC results.

### Region of interest analysis

Because the similarity between the CS+ and the PS was the underlying driver of the classifier’s performance in the regions we revealed (see *Results*), we wanted to know whether the information between these two stimulus classes also changed in higher-level visual areas. To this aim, we wanted to determine whether the resultant classification changes were linked to changes in visual perception information regarding the stimulus categories. Thus, we ran an additional classification and similarity analysis on two regions of interest: the lateral occipital complex and the posterior fusiform gyrus, as prior work has shown that these regions contain category-level information pertaining to animals (Connolly *et al.*, 2012; Dunsmoor *et al.*, 2014). Voxels belonging to these regions in standard MNI space were obtained from the Harvard–Oxford cortical atlas (using 75% probability as a cutoff), and then transformed into subject-space before running the analysis. The training and testing of the LDAC on *t*-score patterns for CS+ vs. PS followed a leave-one-run-out cross-validation scheme at each experimental phase. The cosine analysis was similar, with the exception that all patterns for a given stimulus class in a given experimental phase were averaged together [as only one overall pattern per category is necessary for the computation of representational similarity analysis (Kriegeskorte *et al.*, 2008), see also *Similarity Analysis*] before computing the cosine of the stimulus class vector pairs. Values from both analyses were Fisher-transformed before performing group-level statistics.

## Results

**Fig. 3.**
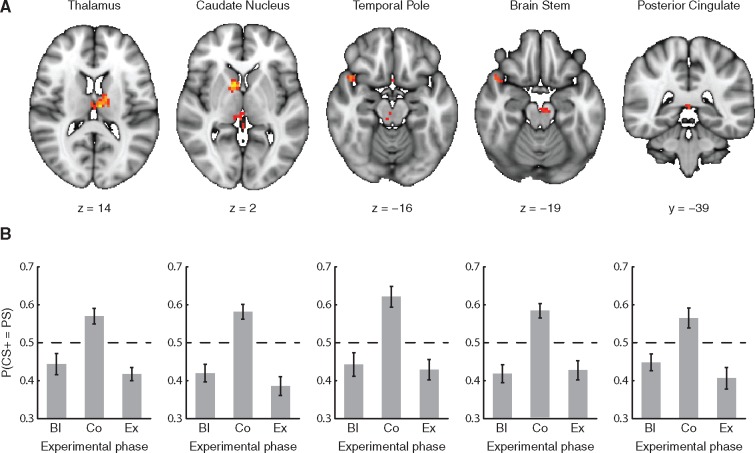
Revealed brain regions and their respective classification performances (A) The five brain regions whose classification profiles showed the predicted inverted-U (see Figure 1). Depicted clusters survived a TFCE-based resampling procedure (see Materials and Methods) and are visualized at z ≥ 2.5758 (*P <* 0.005, FWER) on a standard 1 mm MNI152 brain. For the peak voxel of each of these regions: (B) A visualization of the probability of the classifier categorizing CS+ information as PS information across the three experimental phases (Bl, Co, and Ex) underlying each of the regions in Figure 3A. Note that for all regions, the classifier considered the CS+ as PS in the conditioned phase, but not in the baseline and extinction phases. Error bars represent SEM.

Our main question concerned whether patterns of CS+ became more similar to patterns of PS after fear conditioning. Using a whole-brain searchlight analysis, we tested this question by observing whether the LDAC categorized patterns of CS+ as NS during the baseline and extinction phases while categorizing patterns of CS+ as PS during the conditioned phase. We assessed the LDAC’s performance across all experimental phases at once with a quadratic regression (seeking regions whose quadratic coefficients of the classifier’s outcome yielded an inverted-U, Figure 1, left panel) and a threshold-free cluster enhancement (Smith and Nichols, 2009) resampling procedure for statistical corrections. This procedure identified five regions (Figure 3A, Supplementary Tables S1 and S2) whose decoding performance matched those predictions: the left thalamus [*t*_(20)_ = −7.985, p_FWER_ = 0.011, z(p) = 3.062], right caudate nucleus [*t*_(20)_ =  −9.257, p_FWER_ = 0.002, z(p) = 3.540], right temporal pole [*t*_(20)_ =  −7.817, p_FWER_ = 0.0027, z(p) = 2.782], brain stem [*t*_(20)_ =  −6.136, p_FWER_ = 0.0038, z(p) = 2.669], and posterior cingulate cortex [*t*_(20)_ =  −6.253, p_FWER_ = 0.0038, z(p) = 2.669] (Figure 3B).

**Fig. 4.**
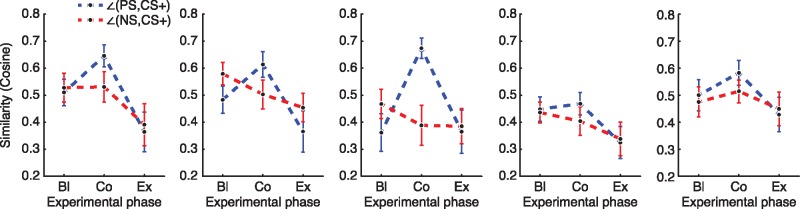
From the peak voxels of the regions of Figure 3A: the cosine of the angle between average stimulus-class vectors across the three experimental phases, representing their similarity, which increased for the CS+ and the PS in most of the regions after conditioning (see Figure 2B). Error bars represent SEM.

**Fig. 5.**
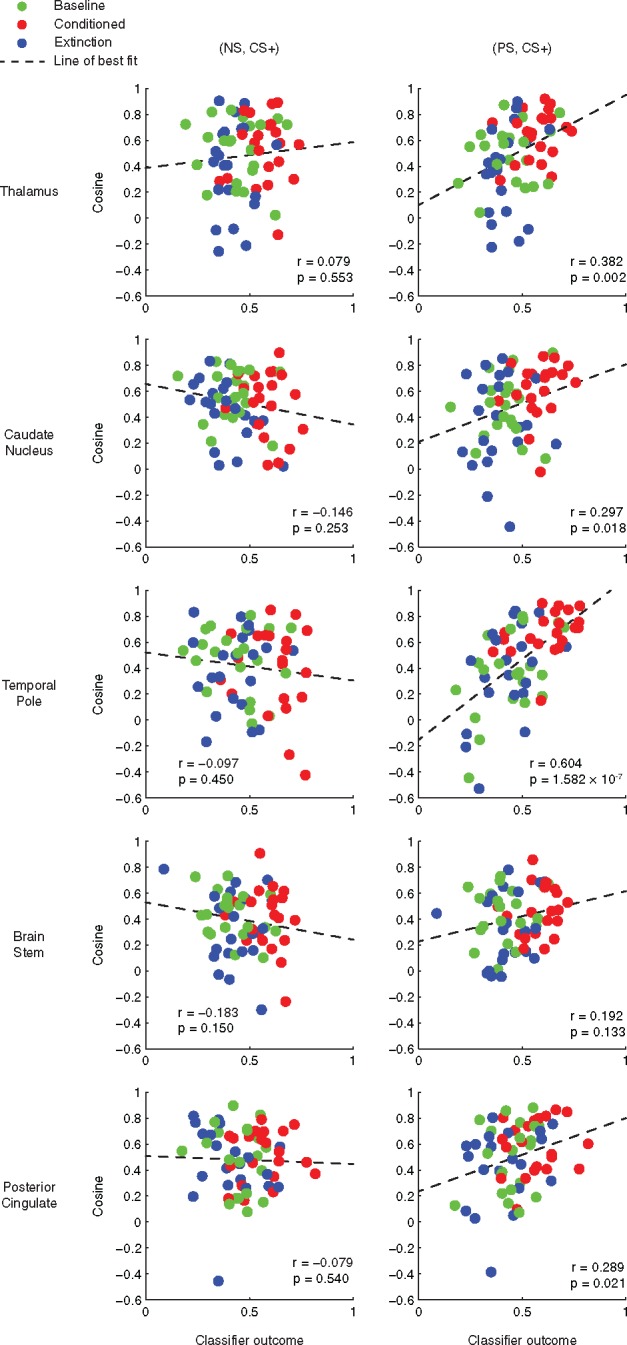
To explore which changes in the representational space potentially steered the classifier’s performance, we correlated each cosine pair from Figure 4 [i.e. either cos(∠_NS, CS+_) or cos(∠_PS, CS+_)] across the three experimental phases) with the respective classification performance from Figure 3B. This analysis yielded positive correlations (Pearson’s r) for four of the five regions (rows) between the classification results and the changing cosine-values of ∠_PS, CS+_ (right column), but not ∠_NS, CS+_ (left column). Dashed lines represent least-squares lines of best fit. These results suggest that mechanisms concerned with representations of fear-concept information underlie these regions, as it is the similarity between the CS+ and the PS (but not the dissimilarity between the CS+ and the NS) that correlates with how the CS+ is categorized.

Such results could explain the effect of fear conditioning by one of three potential models. With respect to the similarity between CS+, NS, and PS information during baseline (Figure 2A), the similarity between CS+ information and PS information could increase (Figure 2B), the similarity between CS+ information and NS information could decrease (Figure 2C), or both (Figure 2D). In order to determine which of these possibilities potentially drove the classifier’s differential performance, we analyzed the similarity (Kriegeskorte *et al.*, 2008) of the categories’ average patterns by measuring the cosine of the internal angle of their vectors from the searchlight surrounding the peak voxel of each region of interest. This analysis yielded higher cosine-values for the angle between the PS and CS+ vectors, cos(∠_PS, CS+_), but not between the NS and CS+ vectors, cos(∠_NS, CS+_), across the three experimental phases (Figure 4, Supplementary Table S3), which demonstrated that, at first glance, the model from Figure 2B best fits the data that the classifier’s performance was driven by changing similarity between CS+ and PS, rather than between CS+ and NS. However, to quantify this link between the changing similarity and the categorization of the CS+’s, we correlated the resulting cosine-values with the classifier’s performance (Figure 5), which revealed, after correcting for multiple comparisons, cos(∠_PS, CS+_) changes correlated with classifier performance in the thalamus (*r* = 0.382, *P <* 0.002) and the temporal pole (*r* = 0.604, *P <* 1.58 × 10^−7^) but not in the caudate (*r* = 0.297, *P <* 0.018), posterior cingulate (*r* = 0.289, *P <* 0.021), or brain stem (*r* = −0.183, *P <* 0.150). Conversely, cos(∠_NS, CS+_) changes did not correlate with classifier performance in any of the regions [thalamus: *r* = 0.079, *P <* 0.553; caudate: *r* = −0.146, *P <* 0.253; temporal pole: *r* = −0.097, *P <* 0.450; cingulate: *r* = −0.079, *P <* 0.540; brain stem: (*r* = 0.192, *P <* 0.021)]. All *P*-values reported in this paragraph, including the FWER threshold of *P <* 0.005, are two-tailed.

**Fig. 6.**
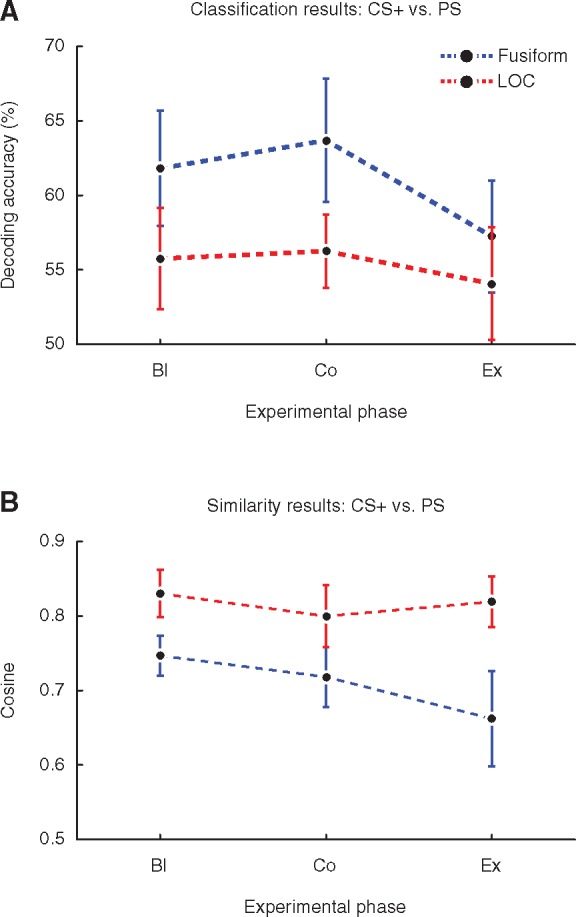
Results from the region-of-interest analysis. (A) Decoding accuracy of CS+ patterns vs. PS patterns within the fusiform gyrus (blue line) and the lateral occipital complex (red line) across the three experimental phases. Thus, fear-conditioning did not hinder classification performance. (B) Cosine values of the angle between CS+ patterns and PS patterns across the three experimental phases. Note that similarity did not increase as a result of conditioning. For both panels, error bars represent 95% confidence intervals.

To discern whether these results reflect changes in abstract fear information or altered perceptual information, we asked whether, as a function of fear-conditioning (and fear-extinction), CS+ information and PS information also become more (and then less) similar to each other in higher-level visual areas. To this end, we performed region-of-interest (ROI) analyses on the lateral occipital complex (LOC) and the fusiform gyrus. The classification analysis of CS+ patterns vs. PS patterns revealed that the LDAC decoded the classes better-than-chance at all experimental phases in both the fusiform (Bl: *t*_(20)_ = 5.03, *P <* 0.00003; Co: *t*_(20)_ = 5.54, *P <* 0.000009; Ex: *t*_(20)_ = 3.13, *P <* 0.0026) and the LOC (Bl: *t*_(20)_ = 2.88, *P <* 0.0046; Co: *t*_(20)_ = 4.29, *P <* 0.0002; Ex: *t*_(20)_ = 1.88, *P <* 0.037; Figure 6A, Supplementary Table S4). The fact that (i) the classifier successfully discriminated between the CS+ and PS at each experimental phase and (ii) we failed to find evidence for either overall differential classifier performance or angular differences across experimental phases (*F*_(2, 40)_ = 2.22, *P <* 0.122 and *F*_(2, 40)_ = 1.47, *P <* 0.242, respectively) or specific to one of the two regions (*F*_(2, 40)_ = 0.78, *P <* 0.464 and *F*_(2, 40)_ = 1.09, *P <* 0.347, respectively) indicates that there were no conditioning-induced changes in perceptual information that could have driven the results in the main analysis.

Following up the LDAC’s successful decoding performance, we wanted to see if there were any underlying changes in the similarity between CS+ and PS patterns. Computing the cosine of the averaged stimulus-class vectors from the baseline, post-conditioning, and extinction phases (Figure 6B, Supplementary Table S4), we did not find evidence that the angle between CS+ patterns and PS patterns differed between the baseline and conditioned phases (fusiform: *t*_(20)_ = 1.297, *P <* 0.21; LOC: *t*_(20)_ = 0.82, *P <* 0.42) or between the conditioned and the extinction phases (fusiform: *t*_(20)_ = 1.294, *P <* 0.21; LOC: *t*_(20)_ =  −0.37, *P <* 0.71).

## Discussion

In this experiment, we sought to investigate patterns of brain activity that reflect abstract information pertinent to the state of fear. By incorporating both a fear-conditioned stimulus and a phobic stimulus into the same experiment, we were able to detect brain regions whose underlying representations reflect commonalities between both types of fear. By pitting three models against each other (Figure 2), our results support the hypothesis depicted in Figure 2B, demonstrating that aversive conditioning leads to a change in the pattern of brain activity such that viewing neutral images of non-threatening animals (e.g. dogs) evokes patterns of brain activity that are more similar to the activity evoked by previously feared stimuli (e.g. spiders), which had not been paired with the aversive stimulus. We show that this change in informational content underlies activity in the left thalamus and the right temporal pole, whereby fear conditioning moves a previously neutral stimulus through the representational space towards a phobic stimulus, and that this movement (and thus similarity of such fear information) can be evaluated via the angle between the local patterns that represent the stimuli.

Our results demonstrate a level of commonality between experimentally induced fear and phobic fear, which is in line with the notion of shared mechanisms for encoding fear memories across fear types (Gross and Canteras, 2012). Such fear-related processes have been linked to the thalamus, for example for context learning in threatening situations (Krout and Loewy, 2000; Carvalho-Netto *et al.*, 2010) or for regulating fear processing in related circuits (Penzo *et al.*, 2015), while the anterior temporal lobe has been associated with emotional memory (Strange and Dolan, 2006), observing actions that express fear (Grèzes *et al.*, 2007), learning emotion information (Todorov and Olson, 2008), and binding higher level emotional and social information (Olson *et al.*, 2007). Our findings encompass both the thalamus and temporal pole, suggesting that fear-related mechanisms (though not necessarily with the same function), which abstract away from specific sources of fear, may underlie information processing in these regions. Additionally, building off the idea that fear is a central state with certain internal representations for motivating specific behavior (Adolphs, 2013), one might posit that our results represent a common aspect of the state of fear. Moreover, our follow-up analysis demonstrated that these changes cannot be attributed to alterations in visual perception information.

Other recent fear-learning studies that employed MVPA have shown that fear conditioning of particular stimuli of a category increases the entire within-category similarity (Dunsmoor *et al.*, 2014) and that patterns of information pertaining to a CS+ are similar to those pertaining to the US (Onat and Büchel, 2015), suggesting that the US guides the generalization of learned fear information and pulls exemplars of a given category closer to one another in the representational space. By using phobic stimuli as reference points for the state of fear (rather than only using a US), we show that informational changes from momentarily acquired fear (to dogs) share commonalities with a more deeply engrained phobic fear (to spiders), which had not been paired with the US. Furthermore, if different types of fear, pre-existing vs. momentarily conditioned, were restricted to entirely different processes, then we would not expect to find brain regions in which CS+ information is categorized as similar to phobia information. Additionally, if fear conditioning resulted from increased visual similarity, then we would have expected patterns of information in visual regions to be more similar to one another and thus indistinguishable. As such, this shifting of a stimulus’ representation toward the representation of a non-conditioned-but-already-fearful category allows us to postulate that the aforementioned brain regions carry common representations, or operate generic mechanisms, for processing information pertinent to the abstract state of fear. This is bolstered by the results from our similarity analysis, which supported the model in which CS+ information became more similar to PS information but did not change with respect to NS information (see Figure 2B).

This perspective sheds new light on the extent to which fear conditioning and extinction change the informational content—pertaining to an abstract state of fear—within a stimulus’ mental representation, thus justifying fear-conditioning as a model for phobic disorders and extinction as a model for exposure therapy. Future directions will involve determining whether this effect generalizes to other types of phobias, establishing where along the information-processing stream this commonality occurs, and examining how medication and psychotherapy alter the underlying mechanisms.

## Supplementary Material

Supplementary Tables
