# Supplementary material for: Relating experimentally-induced fear to pre-existing phobic fear in the human brain
Source: Soc Cogn Affect Neurosci. 2017 Dec 21;13(2):164–72. doi: 10.1093/scan/nsx147 (PMC5827344; doi:10.1093/scan/nsx147)
Supplement: Supplementary Tables [file nsx147_supp.pdf]

Supplementary Information

Region	MNI coordinates (x,y,z)	Quadratic coefficient (mean ± SEM)	t-score	p-value (FWER)
Thalamus	-5, -15, 14	-0.139 ± 0.018	-7.985	0.011
Caudate Nucleus	10, 5, 2	-0.179 ± 0.019	-9.257	0.002
Temporal Pole	46, 14, -16	-0.185 ± 0.024	-7.817	0.0027
Brain Stem	-5, -21, -19	-0.161 ± 0.026	-6.136	0.0038
Posterior Cingulate	1, -39, 2	-0.138 ± 0.022	-6.252	0.0038

Table S1 Descriptive statistics for the quadratic regression analysis at the peak voxel of each of the regions depicted in Figure 1.

Region	Baseline (mean ± SEM)	t-score (p-value)	Conditioned (mean ± SEM)	t-score (p-value)	Extinction (mean ± SEM)	t-score (p-value)
Thalamus	44.33 ± 2.8%	-2.04 (0.027)	56.97 ± 2.1%	3.31 (0.0017)	41.76 ± 1.7%	-4.72 (0.00007)
Caudate Nucleus	41.97 ± 2.3%	-3.28 (0.00186)	58.18 ± 2.0%	4.10 (0.00028)	38.57 ± 2.5%	-4.57 (0.00009)
Temporal Pole	44.28 ± 3.2%	-1.86 (0.039)	62.10 ± 2.8%	4.42 (0.0001)	42.91 ± 2.7%	-2.60 (0.0086)
Brain Stem	41.85 ± 2.4%	-3.39 (0.0015)	58.42 ± 1.9%	4.44 (0.0001)	42.75 ± 0.02.5%	-2.58 (0.0089)
Posterior Cingulate	44.80 ± 2.2%	-2.30 (0.016)	56.50 ± 2.6%	2.49 (0.011)	40.63 ± 2.9%	-3.27 (0.0019)

Table S2 Descriptive statistics for the classification analysis at the peak voxel of each of the regions depicted in Figure 1. All p-values reported here at one-tailed.

	$\cos(\angle_{NS,CS+})$ [mean ± SEM]			$\cos(\angle_{PS,CS+})$ [mean ± SEM]		
Region	Baseline	Conditioned	Extinction	Baseline	Conditioned	Extinction
Thalamus	0.528 ± 0.053	0.531 ± 0.056	0.391 ± 0.077	0.510 ± 0.049	0.645 ± 0.040	0.365 ± 0.073
Caudate Nucleus	0.579 ± 0.042	0.503 ± 0.053	0.454 ± 0.053	0.483 ± 0.051	0.614 ± 0.047	0.366 ± 0.077
Temporal Pole	0.468 ± 0.054	0.388 ± 0.074	0.385 ± 0.066	0.361 ± 0.070	0.674 ± 0.037	0.365 ± 0.080
Brain Stem	0.436 ± 0.038	0.404 ± 0.053	0.339 ± 0.063	0.448 ± 0.045	0.468 ± 0.042	0.324 ± 0.059
Posterior Cingulate	0.475 ± 0.056	0.515 ± 0.042	0.450 ± 0.063	0.500 ± 0.058	0.583 ± 0.046	0.428 ± 0.064

Table S3 Descriptive statistics for the cosine of the angular changes between stimulus class vectors of the searchlight surrounding the peak voxel of the aforementioned regions.

	Decoding accuracy (mean ± SEM)			Cosine (mean ± SEM)		
Region	Baseline	Conditioned	Extinction	Baseline	Conditioned	Extinction
Fusiform gyrus	61.81 ± 2.2%	63.69 ± 2.4%	57.24 ± 2.2%	0.747 ± 0.016	0.718 ± 0.023	0.662 ± 0.037
Lateral occipital complex	55.75 ± 1.9%	56.25 ± 1.4%	54.07 ± 2.2%	0.830 ± 0.018	0.799 ± 0.024	0.819 ± 0.019

Table S4 Descriptive statistics for the classification analysis and the similarity analysis of CS+ vs. PS in the fusiform gyrus and the lateral occipital complex.
